# Sensitivity Analysis of Intracellular Signaling Pathway Kinetics Predicts Targets for Stem Cell Fate Control

**DOI:** 10.1371/journal.pcbi.0030130

**Published:** 2007-07-06

**Authors:** Alborz Mahdavi, Ryan E Davey, Patrick Bhola, Ting Yin, Peter W Zandstra

**Affiliations:** 1 Department of Chemical Engineering and Applied Chemistry, University of Toronto, Toronto, Ontario, Canada; 2 Institute of Biomaterials and Biomedical Engineering, University of Toronto, Toronto, Ontario, Canada; The University of Tokyo, Japan

## Abstract

Directing stem cell fate requires knowledge of how signaling networks integrate temporally and spatially segregated stimuli. We developed and validated a computational model of signal transducer and activator of transcription-3 (Stat3) pathway kinetics, a signaling network involved in embryonic stem cell (ESC) self-renewal. Our analysis identified novel pathway responses; for example, overexpression of the receptor glycoprotein-130 results in reduced pathway activation and increased ESC differentiation. We used a systematic in silico screen to identify novel targets and protein interactions involved in Stat3 activation. Our analysis demonstrates that signaling activation and desensitization (the inability to respond to ligand restimulation) is regulated by balancing the activation state of a distributed set of parameters including nuclear export of Stat3, nuclear phosphatase activity, inhibition by suppressor of cytokine signaling, and receptor trafficking. This knowledge was used to devise a temporally modulated ligand delivery strategy that maximizes signaling activation and leads to enhanced ESC self-renewal.

## Author Summary

Directing stem cell fate requires knowledge of how intracellular signaling pathways integrate environmental stimuli to make decisions to stay as stem cells (self-renew) or to differentiate into specific functional cell types. We developed and validated a computational model of signal transducer and activator of transcription-3 (Stat3) pathway kinetics, a signaling network involved in mouse embryonic stem cell (ESC) self-renewal. Our analysis demonstrates that stem cell fate control is regulated by a distributed set of parameters that positively and negatively regulate Stat3 activation. We further demonstrate that we can take advantage of differences in the timing of signaling pathway activation and inhibition to design a strategy to deliver self-renewal stimuli to stem cells in a more efficient manner. Ultimately, the use of stem cells in biotechnological applications will require an in-depth understanding of how cells integrate diverse environmental stimuli to make cell fate decisions.

## Introduction

Self-renewal is one of the defining characteristics of embryonic stem cells (ESCs) [1]. This fate choice is influenced by ligand–receptor-mediated activation of intracellular signaling pathways. Significant work is being done to understand the signaling proteins and pathways that control self-renewal of ESCs, and an emerging picture is that these pathways influence self-renewal in a context-dependent and temporally modulated manner [2–4]. One such pathway is the Jak/Stat3 (Janus kinase / signal transducer and activator of transcription-3) pathway [5]. Activation of Stat3 by phosphorylation at Tyr-705 results in induction of genetic programs that are sufficient for maintenance of self-renewal in mouse ESCs [6–8]. Understanding how Stat3 activation is controlled may be useful for controlling ESC self-renewal.

Stat3 is activated by a variety of ligands from the interlukin-6 (IL-6)–type family [9]. In mouse ESCs, Stat3 activation results from binding of leukemia inhibitor factor (LIF) to the LIF receptor and glycoprotein-130 (GP130), forming a heterodimeric receptor complex [10,11]. Jak-mediated Src homology-2 (SH2)–domain phosphorylation of receptors leads to Stat3 recruitment to the receptor complex [12], and its Tyr-705 phosphorylation and subsequent nuclear accumulation [13–25]. This pathway is under control of three main inhibitors, protein inhibitor of activated Stat3 (PIAS3), Src-2 homology containing phosphotyrosine phosphatase (SHP2), and suppressor of cytokine signaling (SOCS3). PIAS3 and SHP2 work to reduce Stat3 availability [26] and receptor activation [21,24–26], respectively, and SOCS3, which is under transcription control of Stat3, inactivates activated receptors by binding to GP130 [26,27]. Activation of Stat3 is therefore influenced by a variety of intrinsic pathway components as well as receptor trafficking [28,29]. Understanding how this signaling is controlled presents a challenge which may be best addressed by mathematical modeling [30].

Previous attempts to model the Jak/Stat pathway have either focused on steady state responses or on capturing the transient activation profile of the pathway to understand its kinetics [31–35]. Examining the transient activation profile provides a larger dynamic range of signal activation, and is therefore more amenable to experimental investigation. Although several models have made predictions about the role of different signaling processes in Stat activation, little work has been done to systematically understand how different signaling events contribute to pathway control, and to experimentally validate model predictions. Furthermore, a lack of computationally feasible algorithms for assessing the importance of pathway structure on signaling behavior has prevented an examination of the signaling consequences of all possible intrapathway interactions. To address this limitation, we developed an in silico model of the Jak/Stat3 pathway to computationally screen and classify parameter interactions for their effects on the transient activation profile of Stat3. In performing this global sensitivity analysis, we were able to predict and experimentally verify novel pathway dynamics such as a receptor-concentration–dependent switch in ligand sensitivity. Moreover, this approach allowed us to group pathway interactions into stimulatory- and inhibitory-signaling modules. By focusing our computational analysis on signaling kinetics, we were able to examine the consequence of pathway structure on the kinetics of ligand desensitization. These results led to predictive control of ESC self-renewal by modulating the frequency of ligand stimulation.

## Results

### Simulation of the LIF-Induced Signaling Cascade

**Figure 1:**
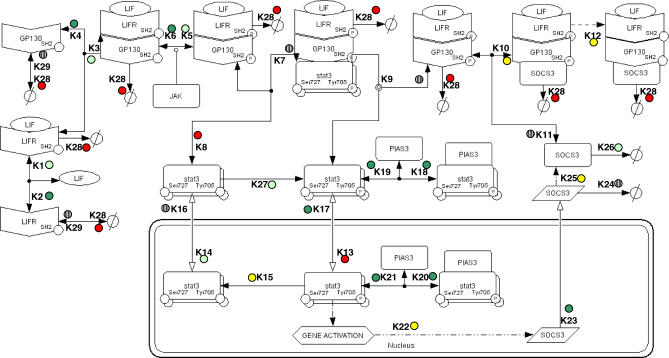
Network Structure of the Jak/Stat3 Pathway Shows Scheme for LIF-induced Activation of Stat3 Closed arrows indicate biochemical reactions, open arrows show transport such as transport into nuclear compartment, and double arrows indicate reversibility. All surface receptors and complexes are internalized similarly, internalized complexes are degraded, and new receptors are constitutively produced. Color-coded dots next to kinetic parameters correspond to grouping of parameters from Figure 5 as explained in text.

LIF-induced activation of Jak/Stat3 pathway was modeled using mass action kinetics for the network structure in Figure 1. The equations for this SBML-compatible [36] system are included in Figure S1, and Table S1 describes the parameters according to MIRIAM standards [37]. Trafficking was assumed to be similar for all surface receptors [38]. Flow cytometry (see Figure S2) demonstrated that surface expression of LIFR and GP130 is unaffected by LIF addition, and that there is a basal level of receptor turnover, consistent with previous findings [26]. Model dependency on starting conditions [39] was determined (see Figure S3), and showed stability over a large range of values.

**Figure 2:**
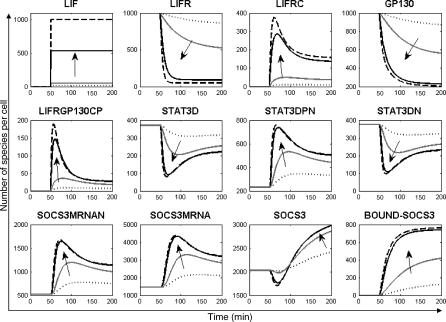
Computational Simulation of Jak/Stat3 Pathway Activation at Different LIF Concentrations Shows Transient Kinetics LIF was added in increasing concentrations of 10, 50, 500, and 1,000 pM in the direction of arrow. LIFRC is the complex of LIFR with LIF, LIFRGP130CP is the phosphorylated heterodimeric complex of LIFR and GP130, STAT3DPN is nuclear Tyr705 phosphorylated Stat3, STAT3DN is nuclear Stat3, SOCS3MRNAN and SOCS3MRNA are the nuclear and cytoplasmic levels of SOCS3 mRNA, and BOUND-SOCS3 is the total number of receptor complexes which are bound to SOCS3. This representative simulation was run for 200 min, and the number of activated proteins per cell are plotted as a function of time.

Simulation results of transient pathway activation as a function of increasing LIF concentration are presented in Figure 2, with exogenous LIF concentration shown in the first panel. In agreement with previous reports [40], there was little difference in Stat3 activation between 500 and 1,000 pM LIF. At 500 pM LIF, LIFR and GP130 receptor complexes formed stabilized dimers in less than ten minutes. Cytoplasmic levels of Stat3 decreased by 4-fold during the first 15 min of LIF addition as Stat3 became phosphorylated and localized to the nucleus, consistent with its observed kinetics in other cells [41]. Even at 10 pM of exogenous LIF, the steady state levels of activated Stat3 were at 60% saturation, and significant changes in SOCS3 induction were observed. Simulations predicted a rapid activation and nuclear localization of Stat3 with a peak at 20–25 min, and a time to equilibrium of 2 h. Next, we experimentally validated model predictions.

### Validation of Model Predicted Kinetics

**Figure 3:**
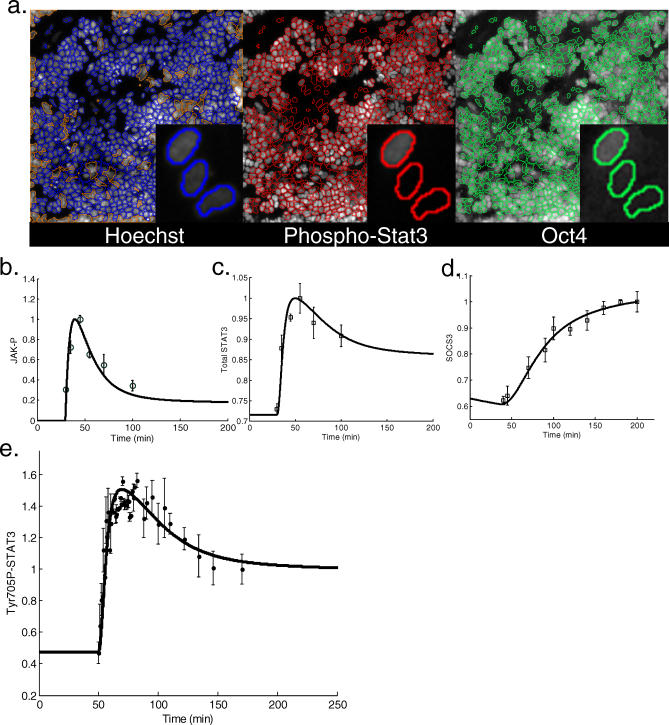
Experimental Validation of Model Output (A) Representative images of mouse ECS for quantitative single cell fluorescence microscopy. Hoechst nuclear stain is used to determine location of nuclei, a nuclear mask is drawn, and average fluorescence levels in the mask are used to determined levels of phosphorylated Stat3 and Oct4. (B–D) Show agreement between model-predicted trends and experimental results of activation profiles of Jak phosphorylation, nuclear retention of Stat3, and SOCS3 induction, respectively. For Jak phosphorylation, nuclear accumulation of Stat3, and SOCS3 induction, both experimental and simulation results were normalized based on the maximum of the activation profiles for each of these proteins. For nuclear accumulation of phosphorylated Stat3 the results were normalized based on a steady state level in 500 pM LIF. (E) The model-predicted activation profile of nuclear retention of Tyr-705 phosphorylated Stat3 is shown in solid line and is in agreement with experimental data points.

Model consistency with experimental output was determined by quantitative image analysis of mouse ESCs [1]. Figure 3A demonstrates how automated fluorescence microscopy combined with single cell image analysis was used to distinguish signaling in undifferentiated ESCs based on expression of stem-cell–maker Octamer-4 binding protein (Oct4). The velocity of receptor activation was determined by assessing the phosphorylation level of Jak (Janus Kinase) during transient LIF stimulation (Figure 3B), and nuclear accumulation of Stat3 (Figure 3C). Excellent agreement was observed between experimental results and model predictions. Analysis of the kinetics of SOCS3 transcription (Figure 3D) and nuclear accumulation of Tyr705 phosphorylated Stat3 (Figure 3E) further supported model predictions. Experimental results validated model predictions, suggesting that the model can be used to investigate the effects of perturbations in signaling parameters on Stat3 signaling kinetics.

### Novel Effects of Receptor Trafficking on Pathway Activation

**Figure 4:**
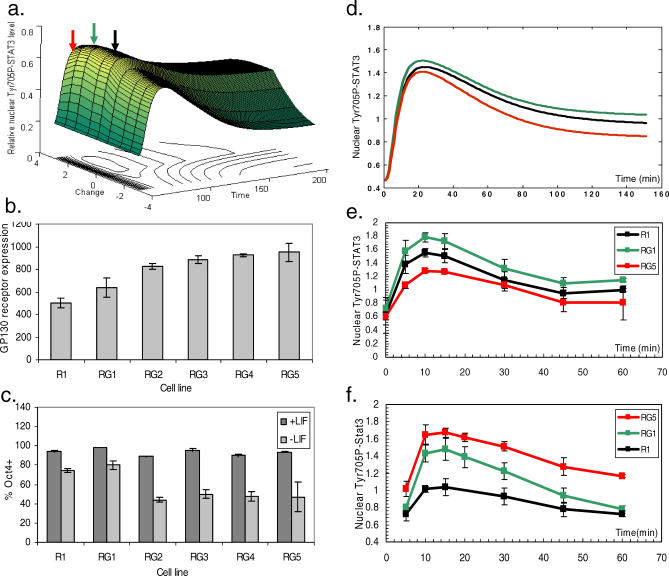
GP130 Overexpressing Cell Lines Show Non-Monotonic Response of Stat3 Activation to Receptor Overexpression (A) The model-predicted surface of Stat3 activation is shown as a function of time and change in production of GP130 receptors. Successive increase in GP130 production as shown by black, green, and red arrows in that order shows a non-monotonic response of Stat3 activation and the presence of a local maximum. This is illustrated by the circle in the contour map on the x–y plane. (B) GP130 overexpressing cell lines RG1–RG5 were developed to express the receptor at varying levels compared with R1 cells, and these cell lines retain Oct4 expression in the presence of LIF (C). (D) Model-predicted trends of GP130 overexpression corresponding to (A) shows normal profile of Stat3 activation (black line), Stat3 activation in slight GP130 overexpression (green line), and significant GP130 overexpression (red line). (E) Experimental results show a consistent trend in LIF-induced Stat3 activation profile using RG1 and RG5 cell lines in comparison with model results in (D). (F) IL-6 stimulation of GP130 overexpressing cell lines shows a dose-dependent increase in Stat3 activation as a function of GP130 overexpression.

To determine the effects of changes in model parameters on Stat3 signaling, we simulated transient pathway behavior over a 40-fold range of values for each parameter to generate Stat3 activation surfaces (see Figure S4 for a complete list). By examining the surfaces for local minima and maxima, which represent non-monotonic effects on Stat3 activation, we found that the only parameter that provided such a response was the production rate of GP130 receptors (Figure 4A). The model predicted a biphasic response; a small increase in receptor expression resulted in an increase in Stat3 activation; while significant receptor overexpression attenuated activation. In silico analysis suggested that transient formation of LIFR and GP130 nonsignaling receptor heterodimers, and sequestering of LIFR by GP130 in the overexpressing cells, is responsible for the observed results, which is consistent with previous reports [28]. Removal of this ligand-independent interaction from the model eliminated the biphasic response to GP130 expression. To validate this prediction, we developed several ESC lines which overexpress the GP130 receptor to varying degrees (Figure 4B and 4C). As predicted by the model (Figure 4D), Stat3 activation increased upon an incremental GP130 overexpression but decreased at higher levels of receptor expression (Figure 4E). To demonstrate that the overexpressed receptors were in fact functional, we examined Stat3 activation in response to IL-6 plus sIL-6R, which signals exclusively through GP130 receptors [25,26]. Cells expressing higher levels of GP130 exhibited higher levels of Stat3 activation upon IL-6 stimulation (Figure 4F), confirming that the overexpressed receptors were functional. As expected from the correlation between Stat3 activation and cell fate processes, modest receptor overexpression appeared to enhance self-renewal in the absence of exogenous LIF, while greater levels of receptor expression increased differentiation (Figure 4B and 4C). In addition to demonstrating the predictive power of this model, these results provide a range of parameter variations over which Stat3 activation is monotonic (see Figure S5). This allowed us to define a range of parameter values over which we could perform a global sensitivity analysis of parameter interactions.

**Figure 5:**
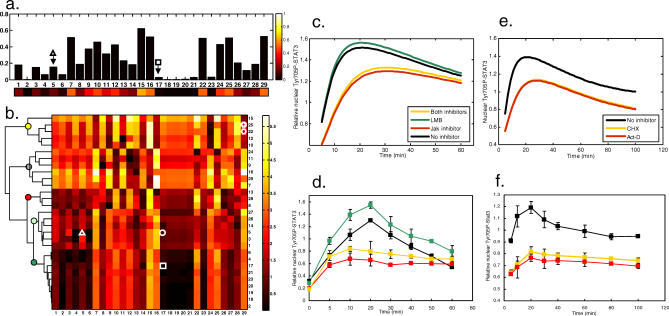
Global Sensitivity Analysis Based on Stat3 Activation (A) Individual rate constants were changed 5-fold, and a Euclidean distance measurement was used to determine the resultant change in the Stat3 activation profile. Normalized Euclidean distance measurements are plotted to show the sensitivity of Stat3 activation to each parameter. (B) Global sensitivity analysis results show changes in Stat3 activation (measured by Euclidean distance) due to 5-fold changes in combinations of parameters pairs, shown along the *x*- and *y*-axis. The sensitivity is represented using color code, with corresponding Euclidean distance values shown in the color bar. Hierarchical clustering (indicated on the left) is based on correlations between rows of rate constants to demonstrate groups of parameters which interact similarly. Clustering was perfomed using Matlab software by Mathworks. Rate constants, which are grouped together, are color-coded using circular dots, and the color code is included in Figure 1. Sensitivity analysis predictions for the Stat3 nuclear export parameter (marked by square), and receptor phosphorylation (marked by triangle), and their interaction (marked by circle) are shown in (C). Sensitivity trends which were predicted in (C) are experimentally verified (D), demonstrating that predicted trends are relevant. (E) Sensitivity analysis trends of interaction of SOCS3 transcription and receptor production (marked by hexagon) and SOCS3 translation and receptor production (marked by cross) are in agreement with experimental results shown in (F).

### Global Sensitivity Analysis Determines Mechanisms of Pathway Control

To determine how different parameters control signal propagation, and thereby impact ESC self-renewal, we performed a global sensitivity analysis (GSA) on Stat3 activation. Model parameters were varied by 5-fold, and the change in the activation profile of Stat3 was quantified using metrics such as Euclidean distance (see Figure S6 for a complete list of metrics used). Sensitivity analysis of parameters in isolation (Figure 5A) showed that the pathway is more sensitive to SOCS3 inhibition than SHP2 or PIAS3. However, parameter interactions have an important role in signal control and must be considered in this context. To do this, a GSA on Stat3 activation was performed for all two-parameter interactions. This approach can be used to cluster different interactions according to their impact on pathway output. Since rows corresponding to parameters group closer together if they interact similarly with other parameters, this approach allowed us to cluster groups of similarly interacting parameters. This approach provides a visual representation that is easy to interpret. The results of this analysis, presented in a clustergram (Figure 5B), show that it is possible to distinguish parameters that contribute to pathway activation (hatched circle) or inhibition (yellow circle), as well as to identify interesting pathway interactions. For example, simultaneously changing the nuclear export rate of Stat3 (K16, all rate constants described in Table S1) and the rate of docking of Stat3 on activated receptors (K7) will influence Stat3 activation more significantly than either of these parameters in isolation or in combination with any other parameters. Our sensitivity analysis demonstrates that nuclear phosphatase activity, inhibition of SOCS3, and Stat3 nuclear export most significantly influence Stat3 activation. These results were unaffected by how much parameters were changed (see Figure S7), and could be averaged over different fold-changes in parameter values (see Figure S8).

To experimentally validate the GSA results, chemical inhibitors were used to specifically target different pathway activation steps (reducing their corresponding rates by 5-fold) and the resultant Stat3 activation profiles were compared with model predictions. For validation, we chose to target nuclear export of Stat3 (K17) and receptor phosphorylation (K5) because of the availability of specific chemical inhibitors and the ability to directly measure the reduction in each rate constant. A CRM-1–dependent nuclear export blocker, Leptomycin-B, was used to reduce phosphorylated-Stat3 nuclear export, and a Jak-specific inhibitor was used to reduce receptor complex phosphorylation. Dose responses of the inhibitors were produced to determine the exact concentrations required to reduce the corresponding rate constants by 5-fold (see Figure S9). As predicted by the model (Figure 5C), experimental results (Figure 5D) show that a 5-fold reduction in surface complex activation by addition of Jak inhibitor will reduce levels of activated Stat3, while a 5-fold reduction in nuclear export of phospho-Stat3 increases levels of activated Stat3. If both inhibitors are used simultaneously, the upstream effect (K5) will dominate the response. We also verified two other sensitivity analysis results involving SOCS3 transcription and receptor production (K22,K29) and SOCS3 translation and receptor production (K25,K29). Model-predicted trends (Figure 5E) were observed experimentally (Figure 5F). In this case, reduction of receptor production decreased the level of Stat3 activation, and reduction of SOCS3 production led to loss of signal attenuation. Knockdown of SOCS3 using siRNA [42] verified that it is responsible for signal attenuation (see Figure S9). Therefore, global sensitivity analysis was used to cluster cell-signaling steps based on intrapathway interactions and identified biologically relevant control nodes of Stat3 activation.

### In Silico Investigation of Desensitization to Ligand Stimulation

**Figure 6:**
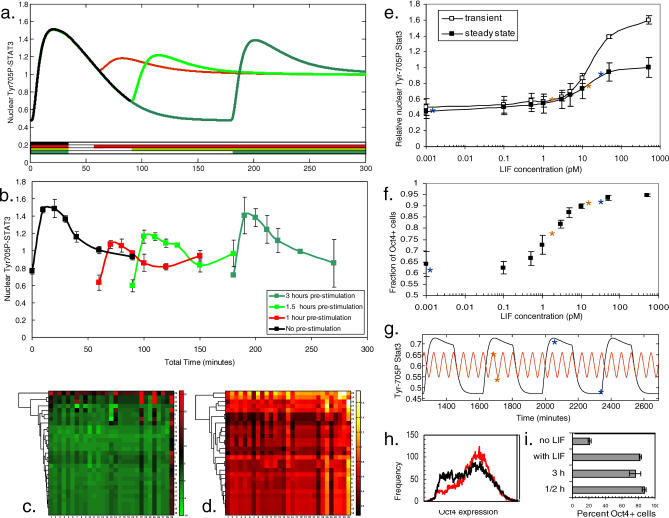
Desensitization of ESCs to LIF Stimulation Can Be Investigated Computationally and Used to Control Self-Renewal (A) Model-predicted trends of desensitization of Stat3 activation to LIF stimulation. Color bar inset shows history of 500 pM LIF stimulations. (B) Experimental results are in agreement with model-predicted desensitization kinetics in (A) and show a gradual loss of desensitization in absence of ligand. (C) Global sensitivity analysis shows parameter interactions which result in right-shift (positive minutes) or left-shift (negative minutes) in restimulation profile of Stat3. Shift in the profile was determined from the maximum of cross-correlation of two consecutive Stat3 activation profiles. (D) Sensitivity analysis clustergram shows Euclidean distance measurement between two consecutive Stat3 activation profiles and distinguishes parameter interactions which influence desensitization by changing the restimulation profile. (E) Experimental results of dose response of Stat3 activation to LIF show the steady state response as well as the peak values of Stat3 activation during transient LIF stimulation. (F) Dose response of Oct4 expression after 72 h at different LIF concentrations. (G) Stat3 activation profiles for 10 pM LIF stimulation with 1-h and 6-h periods in red and blue, respectively. During 6-h cycles, LIF is removed for 3 h during which time a lower Stat3 activation level is observed in comparison with the 1-h period. (H) Histograms of Oct4 expression show that 1-hr period of LIF stimulation (red) maintains a higher percentage of Oct4+ cells than 6-h period of LIF stimulation after 72 h. (I) Oct4 expression of cells maintained for 72 h in different conditions shows that the percentage of Oct4+ cells in the 1-h period is higher than the 6-h period and comparable to 500 pM constant LIF levels.

Desensitization, which is the inability to respond to ligand restimulation [26], may significantly limit cytokine-driven in vitro stem cell propagation [43]. Possible mechanisms of desensitization in the Jak/Stat3 pathway include receptor downregulation, SHP2- or SOCS3-mediated signal attenuation, and differential transport kinetics of STAT3, but the relative importance of each interaction is unknown. As a first step to investigate this response, model-predicted trends of desensitization (Figure 6A) were experimentally verified (Figure 6B), and showed that a minimum time of about 3 h is required after ligand stimulation for the cells to become fully responsive to the readdition of LIF. GSA of two successive Stat3 activation profiles was used to understand what pathway components control desensitization. This method distinguishes between desensitization and inhibition, since both successive activation profiles will be changed by inhibition, but only the reactivation profile is influenced by desensitization. The relative shift of the reactivation profile of Stat3 (Figure 6C), and the sensitivity analysis (Figure 6D), both showed that the decreased production of SOCS3 and a reduction of Stat3 nuclear export (together or independently) could delay the Stat3 reactivation profile. The main determinants of desensitization control, in order of importance, were SOCS3, nuclear phosphatase, and nuclear export of Stat3. Our analysis also suggested that receptor turnover rates influence desensitization.

To determine if Jak/Stat3 pathway desensitization control could impact ESC self-renewal, we experimentally modulated the frequency of LIF-mediated Stat3 stimulation based on the period of cytokine supplementation. Dose responses of Stat3 activation and Oct4 expression at different LIF concentrations identified 10 pM LIF as the minimum concentration that maintains undifferentiated (Oct4+) ESC cells and does not saturate Stat3 activation (Figure 6E and 6F). We reasoned that small modulations of Stat3 above and below this level would reveal sensitivities to desensitization, and thus this concentration of LIF was cycled at two different periods to investigate the impact of desensitization on self-renewal of ESCs. According to our predictions, a 6-h LIF-supplementation period would provide sufficient time (3 h) for Stat3 signaling to reach a minimum and for cells to lose their “memory” of previous LIF levels (Figure 6G). In contrast, a 1-h LIF-supplementation period would result in an increased minimum level of activated Stat3 (Figure 6E–6G) due to desensitization-mediated effects. Consequently, the 1-h LIF cycle should maintain a higher percentage of Oct4+ cells. To experimentally test this prediction, periodic LIF supplementations were performed for a total of 72 h, and the impact of these manipulations on Oct4 expression in ESCs was determined. Results indicated that although total exposure to LIF was the same between these two conditions, the percentage of Oct4+ cells was higher in the 1-h periodic LIF stimulation condition (Figure 6H and 6I). Therefore, the desensitization period may provide windows of opportunity for ESCs to lose memory of previous ligand concentrations and differentiate. Desensitization of the Jak/Stat3 pathway provides an example of how pathways kinetics influences ESC fate choices.

## Discussion

The Jak/Stat3 pathway, which is required for self-renewal of mouse ESCs, was modeled using a deterministic, lumped-parameter, differential-equation–based representation of mass action kinetics. Model-predicted trends of Stat3 activation and nuclear accumulation as well as Jak activation and SOCS3 expression were verified experimentally by quantitative imaging of adherent mouse ESCs. These results provided evidence for biological relevance of model predictions, and led to further investigation of pathway control.

Transient Stat3 activation was used to understand how different signaling events influence pathway kinetics, and grouping of model parameter interactions determined the important intracellular signaling control modules. Sensitivity analysis of model parameters in isolation predicted that significant GP130 overexpression should reduce Stat3 activation. Experimental results of GP130 overexpressing cell lines confirmed predictions, showing decreased Stat3 activation with increased expression of GP130 receptors. Intact biological function of overexpressed receptors was confirmed by IL-6 stimulation of the cells. Based on model results, sequestering of LIFR by GP130 to form a nonsignaling heterodimer, which has been shown to exist transiently [12], can account for this behavior. Higher differentiation was observed in the highest GP130 overexpressing cell lines in the absence of LIF (Figure 4C). This response may be attributed either to reduced Stat3 activation from endogenously produced GP130 ligands [44] or possibly to increased activation of ERK by SHP2 through the GP130 receptors contributing to the response [25,45].

Global sensitivity analysis of the system was performed by considering two parameter interactions; this approach covered the entire solution space of these interactions and provided a computationally feasible method to perform this analysis compared with previous attempts [31]. Clustering (Figure 5B) demonstrated how different parameters interact to yield similar effects. It is noteworthy that sets of inhibitory and stimulatory signaling interactions appeared distributed across the signaling network. Points of sensitivity may represent important nodes of crosstalk between pathways, and nonintuitive pathway behavior could arise from the alteration of seemingly innocuous parameters. These possibilities may in part account for the observation that conserved signaling pathways affect a variety of cell processes in a cell-type and context-dependent manner. A number of other signaling pathways have recently been implicated in ESC self-renewal; this analysis provides a first step toward understanding this signaling crosstalk.

GSA results were verified experimentally by specifically targeting signaling steps for which specific inhibitors were available. The impact of a reduction in the rates of nuclear export of Stat3- and Jak-mediated receptor activation in isolation and in combination were determined experimentally and corroborated model predictions. Interaction of two targets, which clustered together in the GSA, SOCS3 induction and receptor production, was verified, demonstrating that they synergize similarly. Although this sensitivity analysis focused on Stat3 activation due to its importance to self-renewal of mouse ESCs; the same analysis could be performed for any protein in the signal transduction pathway (see Figure S10). Based on GSA, nuclear export of Stat3, nuclear phosphatase activity, and inhibition by SOCS3 were the most sensitive parameters for manipulating pathway output. Although other mechanisms of desensitization, in particular receptor desensitization or differential transport kinetics between phosphorylated and unphosphorylated receptors, are possible, our investigation points out a series of possible mechanisms through which desensitization can occur. More importantly, this approach allows for determination of importance of each mechanism to desensitization. Computational investigation of the dynamics of pathway desensitization to ligand stimulation was a particularly interesting application of GSA. In agreement with previous reports, SOCS3, but neither SHP2 nor PIAS3 kinetics, could account for pathway desensitization [26].

The phenomena of desensitization is an interesting feature of the Jak-Stat signaling pathway, which lends itself to a computational approach for identifying methods of circumventing this inhibitory behavior. To do this, we predicted the effects of two different ligand-dosing frequencies on Stat3 activation and examined their consequences on ESC fate. Although both sets of conditions were exposed to the same overall amount of LIF, the conditions with the LIF cycle period of 1 h retained a higher percentage of Oct4+ cells. Therefore, knowledge of dynamic pathway behavior such as desensitization can be used to identify nonintuitive targets or cell culture manipulations to control stem cell behavior.

In summary, directing stem cell fate requires knowledge of how intracellular signaling can be controlled. To do this, we used a combined computational and experimental approach to study the kinetics of the Jak/Stat3 pathway in mouse ESCs. Although such mass action kinetics models can be improved, for example by including more detailed reaction mechanisms and interpathway interactions, this approach utilized herein provided us with an appropriate representation of the network dynamics and has allowed identification of interesting behaviors and properties of this signaling pathway. Modeling predicted novel effects of signaling (which were experimentally verified), and global sensitivity analysis led us to understand how this pathway is controlled. Based on computational predictions of desensitization kinetics, the self-renewal response of ESCs was modulated by controlling the frequency of ligand stimulation. This approach is useful for optimizing stem cell cultures by providing a ligand delivery regimen based on knowledge of intracellular signaling pathway kinetics. Our approach can be used to find key targets for control of intrapathway kinetics in stem cells. What is now required is to develop direct links between the nuclear concentration of activated Stat3 and cell fate decisions in individual cells. Ultimately, this requires insight into how cells convert graded stimuli into a threshold-based cell fate decision. Adapting the model presented herein to single cell signaling and integrating it with approaches to describe gene regulatory networks will yield a deep understanding of stem cell self-renewal.

## Materials and Methods

### Model development and implementation.

The Matlab software package (The Mathworks, http://mathworks.com) was used for model development and implementation. Signal propagation starts with binding of LIF to its LIFR [10,11]; this is followed by recruitment of the glycoprotein 130 (GP130) receptor to form a stabilized heterodimeric receptor complex on the cell surface. Transient homodimers of LIFR and GP130 are nonsignaling in this system [12]. LIF-mediated heterodimeric receptor complex formation leads to activation of receptor-associated Jaks, which phosphorylate the complementary SH2 domain of both LIFR and GP130 [24,25]. The transcription factor Stat3 then docks on the phosphorylated SH2 domains of the receptor heterodimer and is phosphorylated at Tyr705 by Jak [23]. In this model, we assumed pre-association of Jak with the receptors according to literature [21,22], and we have assumed reversible kinetics as shown by double arrows in Figure 1 [20]. Stat3 is predominantly dimeric [17–19] and cytoplasmic [15,17] in its unphosphorylated form, and translocates to the nucleus upon phosphorylation by using the importin machinery, in particular importin α3–5 [15,24]. In addition to ligand-mediated nuclear import of Stat3 [15,16], there is a basal level of nuclear–cytoplasmic shuttling of unphosphorylated Stat3 [15,17], and dephosphorylation of phosphorylated nuclear Stat3 leads to its CRM-1 dependent nuclear export [14]. We modeled these events by reversible binding of Stat3 to activated receptor complexes (rate constants K7, K8), nonreversible phosphorylation and dissociation of Stat3 from the receptor complex [13] (K9), followed by reversible nuclear translocation of both phosphorylated and unphosphorylated Stat3. We used a set of lumped parameters for all nuclear transport events (K13–17) where import rates are similar [34,38]; however, the export rate of phosphorylated Stat3 is lower, in accordance with previous work [14,41]. We optimized this profile by tuning the export rate constant of phosphorylated Stat3 based on the rise time of its activation profile as determined experimentally. Three important inhibitors of the Jak/Stat3 pathway are also implemented. SOCS3 is under transcription control of Stat3 [26,27], binds to the activated receptor complex of LIF and GP130 by binding to the Tyr974 of the GP130 receptor in the complex, and renders it inactive by removing the ability of Jak to phosphorylate Stat3 [25,26]. Transcription control of SOCS3 by Stat3 and transport rates for the mRNA were modeled by using lumped parameters. Translated SOCS3 protein binds reversibly to the activated herterodimeric receptor complex and renders it inactive, and it dephosphorylates the receptor complex while remaining bound. The second inhibitor of the pathway, PIAS3, has been shown to be present in both nuclear and cytoplasmic compartments, to bind directly to phosphorylated Stat3, is believed not to be under the transcription control of Stat3, and removes the DNA binding ability of phospho-Stat3 [26]. We modeled PIAS3 activity by two reversible binding steps in the cytoplasm and nucleus. Finally, the effect of SHP2, which is the third main inhibitor of the pathway, was captured in the dephosphorylation of the Src-Homology-2 SH-2 domain phosphorylated receptor heterodimer complexes, using rate constant (K6). This is the suggested mode of action of SHP2 in this system [21,24–26]. Trafficking of the receptors GP130 and LIFR is also implemented in the model, and all surface receptors can be internalized with the same rate constant (K28), there is a fixed rate of receptor production using rate constant K29, and internalized complexes do not signal and are degraded [11,28,29]. Using this system, we are able to capture the observed transient kinetics of the Jak/Stat3 pathway.

### ESC culture.

Undifferentiated R1 were maintained in a mixture of 15% fetal bovine serum (FBS) (900–108; Gemini Bio-Products, http://www.gembio.com/), 100 μM β-mercaptoethanol (M6250; Sigma-Aldrich, http://sigmaaldrich.com), 2 mM L-glutamine (25030–081; Invitrogen, http://www.invitrogen.com), 0.1 mM nonessential amino acids (11140–050; Invitrogen), 1 mM sodium pyruvate (11360–070; Invitrogen), 50 U/mL penicillin and 50 μg/mL streptomycin (15140–122; Invitrogen) in high-glucose Dulbecco's Modified Eagle's Medium (DMEM) supplemented with 500 pM murine leukemia inhibitory factor (mLIF; ESG11–7; Chemicon, http://www.chemicon.com), on 0.2% gelatin-coated tissue-culture flasks. Single-cell suspensions were obtained by incubating the cells with 0.25% Trypsin-EDTA (25200–106; Gibco, http://www.invitrogen.com) for 3 min, and 1 × 10^6^ cells were plated in a T25 between subsequent passages, using only cells from passages 15 to 30.

### Transient LIF stimulation assays.

In order to obtain adherent cells, special optic flat-bottom, clear 96-well plates (3614; Corning, http://www.corning.com) were precoated with 1% Fibronectin (F1141; Sigma-Aldrich) in 0.02 % Gelatin at 37 °C and 5% CO_2_ for 24 h. Single-cell suspension of R1 ESCs was obtained as outlined above. Cells were plated in 200 ul per well of ESC culture media at a density of 1.5 × 10^4^ cells per well of a 96-well plate, spun using appropriate plate holders at 220 G for 5 min, and incubated at 37 °C and 5% CO_2_ for 4 h in order to allow cell attachment. Thereafter, media was changed to ESC culture media without LIF and with FBS replaced with 15% knockout serum replacement (KO media) (10828–028; Invitrogen), and cells were incubated in this media at 37 °C and 5% CO_2_ for 24 h before LIF stimulation. LIF stimulation was performed by adding KO media containing 500 pM LIF at predefined time points and fixing the cells at time zero with 3.7% formaldehyde in phosphate-buffered saline (PBS). For inhibitor studies, the inhibitors were added at the appropriate concentration in KO media at 200 ul/well for 4 h before LIF stimulation. Jak Inhibitor-1(420099; Calbiochem, http://www.merckbiosciences.co.uk) was used at concentration 5 nM as determined by dose response curves and Leptomycin-B (L2913, Sigma Aldrich) was used at concentration 10 ng/ml (see Figure S9 for dose responses), with appropriate controls containing equal concentration dimethyl sulfoxide (DMSO) (D2650; Sigma-Aldrich).

### High-content single-cell fluorescence microscopy of adherent ESCs.

For intracellular staining, the cells are permeablized with 100 μl per well methanol for 3 min at room temperature and washed three times with 200 μl per well of PBS. To reduce nonspecific binding of antibody, 200 ul of 10% FBS in PBS is added to each well, and the plate was kept at 4 °C for 24 h. Antibody dilutions were performed in the same FBS in PBS composition. The following primary antibodies were used with 24-h incubation: anti-phospho-Stat3 (tyr705) (9131; Cell Signaling Technology, http://www.cellsignal.com/), anti-Oct3 (Oct4) (611203; BD Bioscience, http://www.bdbiosciences.com/), anti-phospho-Jak2 (Tyr1007–1008) (3771; Cell Signaling technology), anti-LIFR (Sc659-Santa Cruz Biotechnology, http://www.scbt.com/), anti- GP130 (Sc656-Santa Cruz Biotechnology), and anti-SOCS3 (Sc9023-Santa Cruz Biotechnology). The following secondary antibodies were used with 2-h incubation: AlexaFluor 488 (A-11034), and AlexaFluor 546 (A-11030; Molecular Probes, http://www.probes.invitrogen.com). Hoechst nuclear dye (B2261; Sigma-Aldrich) was used at 0.1 ug/mL.

Cells were scanned using the ArrayScan VTI automated fluorescent microscope (Cellomics, http://www.cellomics.com/). Image analysis was performed using the vHSC View software provided by Cellomics. Object selection was performed based on Hoechst staining of nuclei, and images of appropriate channels (for example, Oct4 and Phospho-Stat3) were analyzed to determine the average pixel intensity for each cell. Oct4 subpopulations were determined by fitting two Gaussian distributions (OriginPro 7.5; OriginLab, http://www.originlab.com/) to log-transformed data of Oct4 fluorescence. All experiments were performed at least in triplicate, and at least 30,000 cells were imaged per data point.

### Flow cytometry and receptor expression assays.

Cells were fixed after LIF addition at appropriate time points (see Figure S2) using Reagent 1 containing 5.5% formaldehyde (IM2389, Immunotech, http://www.beckmancoulter.com) in 2% FBS Hank's Buffered Salt Solution (HBSS) (14175–095-Gibco) for 15 min. Permeablization (in the case of permeablized cells) was done in Reagent 2 containing 0.1% sodium azide (IM2389, Immunotech) for 5 min, followed by incubation with the appropriate primary antibody for another 15 min. Primary antibodies that were used are listed above, and the secondary antibodies that were used are PE anti-mouse IgG1 (550083; BD Bioscience) and Goat-anti-rabbit FITC (0833; Immunotech).

### Development of GP130 overexpressing cell lines.

GP130 cDNA was provided by Kishimoto et. al [41]. The PCR-amplified fragment was subsequently inserted into the pCAG-GFP vector provided by Dr. J. Draper [46] using the Xho1 and Not1 sites. The cloned product was sequenced. R1 ESCs were transfected by electroporation, and stable transfectants were selected using Puromycin resistance. Five cell lines (RG1–RG5) were expanded and cell-surface GP130 overexpression was confirmed by both flow cytometry and Cellomics imaging.

### SOCS3 siRNA construction and transfection.

siRNA transfections were performed using Lipofectamine transfection reagent (18342–012; Invitrogen). siRNA for murine SOCS3 (sense, 5′-GGAGCAAAAGGGUCAGAGGtt-3′; antisense, 5′-CCUCUGACCCUUUUGCUCCtt-3′) [42] was custom-manufactured (16211; Ambion, http://www.ambion.com). Transfection was performed on adherent cells in KO media without antibiotics, in a volume of 200 ul/well containing 160 ul of plating media, 1–50 pMol of siRNA, and 0.3–0.8 ul of Lipfectamine reagent. After dose response, the optimum condition was found to be 11.4 pmol of siRNA in 200 μl of plating media containing 0.4 μl of Lipfectamine, which was made according to manufacturer instructions (18342–012; Invitrogen). Cells were plated in the transfection reagent 24 h before readout.

## Supporting Information

Figure S1 Diagrammatic Representation of the Signal Network Structure of the Jak/Stat3 Pathway and Mathematical Representation (78 KB DOC)

Figure S2 Flow Cytometry Results of Receptor Expression (227 KB DOC)

Figure S3 Dependency of Model Output on Initial Numbers of Proteins and Receptors (98 KB DOC)

Figure S4 Stat3 Surface Response to Parameter Changes (2.3 MB DOC)

Figure S5 Stat3 Activation Surfaces for Two-Parameter Interactions (877 KB DOC)

Figure S6 Calculating Changes in Activation Profile of Stat3 (42 KB DOC)

Figure S7 Sensitivity Analysis Results Are Independent of Fold Changes in Parameters (58 KB DOC)

Figure S8 Sensitivity Analysis for All Possible Parameter Changes (117 KB DOC)

Figure S9 Dose Responses of Inhibitors (37 KB DOC)

Figure S10 Sensitivity Analysis for Each Protein in the Pathway (129 KB DOC)

Table S1 Rate Constants and Parameters Used in the Mathematical Model (211 KB DOC)

## Abbreviations

ESC: embryonic stem cells
FBS: Fetal Bovine Serum
GP130: glycoprotein-130
GSA: global sensitivity analysis
IL-6: interlukin-6
Jak/Stat3: Janus kinase / signal transducer and activator of transcription
KO media: knockout serum replacement
LIF: leukemia inhibitory factor
LIFR: leukemia inhibitory factor receptor
Oct4: Octamer-4 binding protein
PIAS3: protein inhibitor of activated Stat3
SHP2: Src-2 homology containing phosphotyrosine phosphatase
SOCS3: suppressor of cytokine signaling
Stat3: signal transducer and activator of transcription-3
