# Supplementary material for: Sensitivity Analysis of Intracellular Signaling Pathway Kinetics Predicts Targets for Stem Cell Fate Control
Source: PLoS Comput Biol. 2007 Jul 6;3(7):e130. doi: 10.1371/journal.pcbi.0030130 (PMC1913098; doi:10.1371/journal.pcbi.0030130)
Supplement: Supplementary file 1 [file pcbi.0030130.sg001.doc]

**Supplementary Figure 1**

**Diagrammatic representation of the signal network structure of the JAK/STAT3 pathway and mathematical representation**

**Y1**

**Y2**

**Y3**

**Y4**

**Y5**

**Y6**

**Y7**

**Y5**

**Y19**

**Y8**

**Y9**

**Y17**

**Y10**

**Y16**

**Y15**

**Y14**

**Y11**

**Y12**

**Y8**

**Y18**

**Y13**

Mathematical representation of the pathway:

$$\begin{aligned} \frac{\text{dy}(1)}{\text{dt}}=(-K_{1}\cdot\text{LIF}-K_{\text{28}})\cdot y(1)+K_{2}\cdot y(2)+K_{\text{29}} \\ \frac{\text{dy}(2)}{\text{dt}}=K_{1}\cdot\text{LIF}\cdot y(1)-(K_{2}+K_{3}\cdot y(3)+K_{\text{28}})\cdot y(2)+K_{4}\cdot y(4) \\ \frac{\text{dy}(3)}{\text{dt}}=(-K_{3}\cdot y(2)-K_{\text{28}})\cdot y(3)+K_{4}\cdot y(4)+K_{\text{29}} \\ \frac{\text{dy}(4)}{\text{dt}}=K_{3}\cdot y(3)\cdot y(2)-(K_{4}+K_{5}+K_{\text{28}})\cdot y(4)+K_{6}\cdot y(5) \\ \frac{\text{dy}(5)}{\text{dt}}=K_{5}\cdot y(4)-(K_{6}+K_{7}\cdot y(8)+K_{\text{10}}\cdot y(\text{16})+K_{\text{28}})\cdot y(5)+(K_{8}+K_{9})\cdot y(6)+K_{\text{11}}\cdot y(7) \\ \frac{\text{dy}(6)}{\text{dt}}=K_{7}\cdot y(5)\cdot y(8)-(K_{8}+K_{\text{28}}+K_{9})\cdot y(6) \\ \frac{\text{dy}(7)}{\text{dt}}=K_{\text{10}}\cdot y(5)\cdot y(\text{16})-(K_{\text{11}}+K_{\text{12}}+K_{\text{28}})\cdot y(7) \\ \frac{\text{dy}(8)}{\text{dt}}=(-K_{7}\cdot y(5)-K_{\text{14}}-K_{\text{27}})\cdot y(8)+K_{8}\cdot y(6)+K_{\text{16}}\cdot y(\text{12}) \\ \frac{\text{dy}(9)}{\text{dt}}=K_{\text{27}}\cdot y(8)-(K_{\text{18}}\cdot y(\text{17})+K_{\text{13}})\cdot y(9)+K_{\text{19}}\cdot y(\text{10})+K_{\text{17}}\cdot y(\text{11})+K_{9}\cdot y(6) \\ \frac{\text{dy}(\text{10})}{\text{dt}}=K_{\text{18}}\cdot y(\text{17})\cdot y(9)-K_{\text{19}}\cdot y(\text{10}) \\ \frac{\text{dy}(\text{11})}{\text{dt}}=K_{\text{13}}\cdot y(9)+K_{\text{21}}\cdot y(\text{13})-(K_{\text{17}}+K_{\text{15}}+K_{\text{20}}\cdot y(\text{18}))\cdot y(\text{11}) \\ \frac{\text{dy}(\text{12})}{\text{dt}}=K_{\text{15}}\cdot y(\text{11})-K_{\text{16}}\cdot y(\text{12})+K_{\text{14}}\cdot y(8) \\ \frac{\text{dy}(\text{13})}{\text{dt}}=K_{\text{20}}\cdot y(\text{18})\cdot y(\text{11})-K_{\text{21}}\cdot y(\text{13}) \\ \frac{\text{dy}(\text{14})}{\text{dt}}=K_{\text{22}}\cdot y(\text{11})-K_{\text{23}}\cdot y(\text{14}) \\ \frac{\text{dy}(\text{15})}{\text{dt}}=K_{\text{23}}\cdot y(\text{14})-(K_{\text{24}}+K_{\text{25}})\cdot y(\text{15}) \\ \frac{\text{dy}(\text{16})}{\text{dt}}=K_{\text{25}}\cdot y(\text{15})-(K_{\text{26}}+K_{\text{10}}\cdot y(5))\cdot y(\text{16})+K_{\text{11}}\cdot y(7) \\ \frac{\text{dy}(\text{17})}{\text{dt}}=-K_{\text{18}}\cdot y(9)\cdot y(\text{17})+K_{\text{19}}\cdot y(\text{10}) \\ \frac{\text{dy}(\text{18})}{\text{dt}}=-K_{\text{20}}\cdot y(\text{11})\cdot y(\text{18})+K_{\text{21}}\cdot y(\text{13}) \\ \frac{\text{dy}(\text{19})}{\text{dt}}=K_{\text{12}}\cdot y(7)-K_{\text{28}}\cdot y(\text{19}) \end{aligned}$$
