# Supplementary material for: Sensitivity Analysis of Intracellular Signaling Pathway Kinetics Predicts Targets for Stem Cell Fate Control
Source: PLoS Comput Biol. 2007 Jul 6;3(7):e130. doi: 10.1371/journal.pcbi.0030130 (PMC1913098; doi:10.1371/journal.pcbi.0030130)
Supplement: Supplementary file 2 [file pcbi.0030130.sg002.doc]

**Supplementary Figure 2**

**Flow cytometry results of receptor expression**

**Flow cytometry results of receptor expression**

Flow cytometry results show the changes that occur in levels of LIFR and GP130. Cells are incubated with either 500 pM LIF or the 50ug/ml of translational inhibitor Cycloheximide as indicated on the bottom of the histograms, for the duration of time indicated on the left. The controls are in solid black histograms on top, and the grey histogram depicts the levels of LIFR and GP130 at time t=0, with no treatment. For all other timepoints, the histogram for t=0 is shown in grey for a basis of comparison to the treated sample represented in black outline. Treatment of cells with LIF causes no statistical change in receptor numbers for both LIFR and GP130 receptors by the 70 minute time-point. Treatment of cells with translation inhibitor Cycloheximide causes a persistent decrease in GP130 levels after treatment with Cycloheximide, indicative of basal levels of receptor production as well as internalization/degradation in the absence of cytokine (LIF) stimulation.

A.B. Percentage of live cells and Oct4 levels are assessed at the end of the time course experiment to demonstrate cell viability.

C. 7AAD staining is used to show the percentage of live cells, 97.4%, at the end of the experiment. Gate M is set using a live cell control and gate K is set using a dead cell control using all events captured, data not shown for 7AAd controls. Live cell count shows that cells survive treatment with Cycloheximide for the 1 hour duration of the experiment in KO serum replacement in suspension at 37°Celcius.

D. Oct4 staining shows that the 7AAD negative cells are 88.5% Oct4-high, gating is set using appropriate controls using FITC goat anti mouse 2nd abs. This demonstrates that the results for receptor expressions are obtained from Oct4-high (undifferentiated) live cells.

E. Changes in GP130 receptor numbers are determined by normalizing the mean fluorescence of the GP130 histograms for each time point to the un-treated sample. Percentage of GP130 remaining from t=0 is plotted as a function of time, showing that half life of GP130 is about 70 minutes on the surface of these cells; and is independent of presence/absence of LIF.
