# Supplementary material for: Sensitivity Analysis of Intracellular Signaling Pathway Kinetics Predicts Targets for Stem Cell Fate Control
Source: PLoS Comput Biol. 2007 Jul 6;3(7):e130. doi: 10.1371/journal.pcbi.0030130 (PMC1913098; doi:10.1371/journal.pcbi.0030130)
Supplement: Supplementary file 3 [file pcbi.0030130.sg003.doc]

**Supplementary Figure 3**

**Dependency of model output on initial numbers of proteins and receptors**

Changes in the initial number of proteins show that increases in Stat3 numbers result in a dose dependant increase in the transient activation profile of Stat3 (nuclear retention of Tyr-705 phosphorylated Stat3 upon 500 pM LIF simulation).

Direction of arrows indicates direction of increase in the initial number of proteins.

The total number of initial proteins which were used for simulations is 1000 LIFR and GP130 receptors, and 1000 Stat3 proteins. All other species were set to zero, and were allowed to reach steady state (in the absence of LIF) before LIF stimulation.

For each plot, Stat3 activation (nuclear accumulation of phosphorylated stat3) on the y-axis is plotted as a function of time on the x-axis. Decrease in the level of Stat3 activation results form increased PIAS3 proteins or decrease in initial number of LIFR or GP130 receptors. Increase in the initial number of SOCS3 proteins also shows a decrease in Stat3 activation, however since SOCS3 is under transcriptional control of Stat3, initial numbers of SOCS3 proteins do not make a change to the activation profile of Sat3 as steady state is achieved before each stimulation with LIF.

**Normalized Activated STAT3**

**Increase Stat3**

**Increase PIAS3**

**Decrease LIFR**

**Decrease GP130**

**Increase SOCS3**

Time (minutes)
