# Supplementary material for: Sensitivity Analysis of Intracellular Signaling Pathway Kinetics Predicts Targets for Stem Cell Fate Control
Source: PLoS Comput Biol. 2007 Jul 6;3(7):e130. doi: 10.1371/journal.pcbi.0030130 (PMC1913098; doi:10.1371/journal.pcbi.0030130)
Supplement: Supplementary file 4 [file pcbi.0030130.sg004.doc]

**Supplementary Figure 4**

**Stat3 surface response to parameter changes.**

Surface plots represent level of nuclear Try-705 phosphorylated Stat3 which is activated by addition of 500 pM LIF at 50 minutes (time represented on the x-axis). Each parameter in the model was changed over a 40 fold range (shown in Log scale on the y-axis), and the result is relative Stat3 activation on the z-axis. The contour map in the x-y plane shows the position of local minima and maxima. The only parameter which provided a biphasic response (local maxima) was GP130 production rate constant. In this analysis, in order to show the effect of changes in production rate of GP130 receptors, we designated this rate constant by K_30_.

| **K1**   | **K2**   |
| --- | --- |
| **K3**   | **K4**   |
| **K5**   | **K6**   |

| **K7**   | **K8**   |
| --- | --- |
| **K9**   | **K10**   |
| **K11**   | **K12**   |
| **K13**   | **K14**   |
| **K15**   | **K16**   |
| **K17**   | **K18**   |
| **K19**   | **K20**   |
| **K21**   | **K22**   |
| **K23**   | **K24**   |
| **K25**   | **K26**   |
| **K27**   | **K28**   |
| **K29 (LIF production)**   | **K30 (GP130 production)**   |
