# Supplementary material for: Sensitivity Analysis of Intracellular Signaling Pathway Kinetics Predicts Targets for Stem Cell Fate Control
Source: PLoS Comput Biol. 2007 Jul 6;3(7):e130. doi: 10.1371/journal.pcbi.0030130 (PMC1913098; doi:10.1371/journal.pcbi.0030130)
Supplement: Supplementary file 5 [file pcbi.0030130.sg005.doc]

**Supplementary Figure 5**

**Stat3 activation surfaces for 2-parameter interactions**

Parameters were changed by [1/5,1/2.5,1,2.5,5] fold in pairs and the 20 minute time point value of Stat3p activation was plotted over all 25 combinations of parameter change for each pair of parameters. For example for K1, the interaction between K1 and K2 is the following surface:

Where 1&2 represents interaction of K1 and K2. The x-y plane of the surface plot shows the contour map of the surface.

K1:

K2:

K3:

K4:

K5:

K6:

K7:

K8:

K9:

K10:

K11:

K12:

K13:

K14:

K15:

K16:

K17:

K18:

K19:

K20:

K21:

K22:

K23:

K24:

K25:

K26:

K27:

K28:

K29:
