# Supplementary material for: Sensitivity Analysis of Intracellular Signaling Pathway Kinetics Predicts Targets for Stem Cell Fate Control
Source: PLoS Comput Biol. 2007 Jul 6;3(7):e130. doi: 10.1371/journal.pcbi.0030130 (PMC1913098; doi:10.1371/journal.pcbi.0030130)
Supplement: Supplementary file 6 [file pcbi.0030130.sg006.doc]

**Supplementary Figure 6**

**Calculating changes in activation profile of Stat3**

**Time (min)**

**Removal of Endogenous Effects for Sensitivity Analysis**

**Original profile**

**Changed profile**

**Original profile**

**Changed profile**

**First**

**Transient**

**Second**

**Transient**

**Cytokine**

**+**

**-**

**+**

**-**

**Time(min)**

**Determination of Sensitivity Based on Transient Re-stimulation**

Comparison of original Stat3p profile (no changes in rate constant) with the changed profile (after parameter changes) is shown.

The following equations are used for comparison:

**Correlation coefficient:**

$$r=\frac{n\sum xy-\sum x\sum y}{\sqrt{\left[n\sum x^{2}-(\sum x)^{2}\right]\cdot\left[n\sum y^{2}-(\sum y)^{2}\right]}}$$

**Euclidean distance:**

$$d=\sqrt{\sum_{i=1}^{n} \left|x_{i}-y_{i}\right|^{2}}$$

where n is the size of the signal (for example 400 time points), and x and y are the two profiles being compared.
