# Supplementary material for: Sensitivity Analysis of Intracellular Signaling Pathway Kinetics Predicts Targets for Stem Cell Fate Control
Source: PLoS Comput Biol. 2007 Jul 6;3(7):e130. doi: 10.1371/journal.pcbi.0030130 (PMC1913098; doi:10.1371/journal.pcbi.0030130)
Supplement: Supplementary file 7 [file pcbi.0030130.sg007.doc]

**Supplementary Figure 7**

**Sensitivity analysis results are independent fold changes in parameters**

To demonstrate that the result of sensitivity analysis is not dependant on the fold change in parameters, the sensitivity analysis results of 5 as well as 2.5 fold change in each parameter are represented by correlation coefficient following by Euclidean distance for each parameter. Although the actual value of Euclidean distance measurements are different sine the parameter changes 5 and 2.5 fold, the relative changes are conserved within each sensitivity analysis, hence the patterns are conserved between 2.5 and 5 fold changes.

5X

2.5 X
