# Supplementary material for: Sensitivity Analysis of Intracellular Signaling Pathway Kinetics Predicts Targets for Stem Cell Fate Control
Source: PLoS Comput Biol. 2007 Jul 6;3(7):e130. doi: 10.1371/journal.pcbi.0030130 (PMC1913098; doi:10.1371/journal.pcbi.0030130)
Supplement: Supplementary file 8 [file pcbi.0030130.sg008.doc]

**Supplementary Figure 8**

**ensitivity analysis for all possible parameter changes**

For the 5 fold change sensitivity analysis, the results are obtained for 5 fold increase parameters as well as 5 fold decrease in parameters and all combinations of these changes. A,B represent 5 fold increase and decrease of both parameters respectively. C represents 5 fold increase in 1 parameter and 5 fold decrease in the other parameter (white line indicates that the pattern is not symmetric along K_i,i_). Combination of all the sensitivities is represented in D, which shows that the effects are not additive (otherwise the results of parameter changes would cancel out, and all the values would be similar in D).

A. 5 fold increase in both paramters
B. 5 fold decrease in both parameters
C. 5 fold increase in parameter1, 5 fold decrease in parameter 2
D. Averages sensitivity of all changes
