# Supplementary material for: Sensitivity Analysis of Intracellular Signaling Pathway Kinetics Predicts Targets for Stem Cell Fate Control
Source: PLoS Comput Biol. 2007 Jul 6;3(7):e130. doi: 10.1371/journal.pcbi.0030130 (PMC1913098; doi:10.1371/journal.pcbi.0030130)
Supplement: Supplementary file 9 [file pcbi.0030130.sg009.doc]

**Supplementary Figure 9**

**Dose responses of inhibitors**

Each chemical inhibitor is added in varying concentration in K0 media and the cells are incubated with the inhibitor for 3 hours before imaging. Dose response of inhibitors shows that 10 nM of Jak inhibitor will reduce JAK activation by 5 fold.

The nuclear export blocker LMB was added in increasing concentrations, and total amount of nuclear Stat3p was determined. Based on the change in Stat3p as a function of total amount of Stat3p (the amount at the highest concentration of LMB), the fold change in the ratio of internal to external Stat3 was determined, a concentration of 10 ng/ml of LMB reduces nuclear export by five fold. This is based on the dynamic range of this response. Therefore the change in the internalization was used as a measure of change in the rate of nuclear export. Since the ratio of the import rate constant to export rate constant is to the first approximation the same as the ratio of the nuclear to cytoplasmic levels of Stat3p, based on the ratio of internal to external Stat3p we can determine that at nuclear Stat3p level of 0.91 the export rate constant is changed by about five fold. The ratio can be determined by (1-n)/n, n being the internalized fraction.

Dose response of actinomycin-D show indicate a 5 fold reduction in SOCS3 transcription at 15 ug/ml, and stops transcription at 50 ug/ml. Dose response of Cycloheximide shows a 5 fold reduction in translation of SOCS3 at 20 ug/ml.

SOCS3 siRNA shows SOCS3 has an inhibitor effect on Stat3 activation:

R1 cells were incubated with varying concentrations (in pM) of the siRNA as indicated on the legend. Stat3 activation was measured at 25 minutes during the peak of the activation profile as well as at 60 minutes. Trends of shift in 30 to 60 minute levels shows a dose dependant increase in Stat3 activation in increasing siRNA concentrations. Stat3 activation was normalized to the steady state 500 pM LIF concentration level.
