# Supplementary material for: Sensitivity Analysis of Intracellular Signaling Pathway Kinetics Predicts Targets for Stem Cell Fate Control
Source: PLoS Comput Biol. 2007 Jul 6;3(7):e130. doi: 10.1371/journal.pcbi.0030130 (PMC1913098; doi:10.1371/journal.pcbi.0030130)
Supplement: Supplementary file 10 [file pcbi.0030130.sg010.doc]

**Supplementary Figure 10**

**Sensitivity analysis for each protein in the pathway**

Three measurements of change are used to show the sensitivity for each protein in the pathway. For example, for Stat3 activation which is discussed in detail in this paper, STAT3DPN (nuclear phospho-Stat3 in its dimeric form) is used. However here we show that the same approach can be used to determine the sensitivity of the system based on each protein in the signal transduction pathway.

Euclidean distance and correlation coefficient shows changes in shape.

Euclidean distance: Euclidean distance measurement shows sensitivity of each protein in the signal transduction pathway to changes in rate constants. Higher values of Euclidean distance indicate more change and higher sensitivity.

Correlation coefficient:

Correlation coefficient measures similarity of shape. Higher values of correlation coefficient indicate more similarity; therefore low values of correlation coefficient are indicative of elevated sensitivity to rate constant changes.

Local maxima and minima measurement for sensitivities:

A simple measure of change in each protein is the value of maximum or minimum in the transient activation profile. In the case of Stat3 the maximum would represent the peak of the activation profile. Since some proteins decrease during the activation profile, the minimum values are used, therefore a circle is used to show if the minimum or maximum value is being represented. Interestingly for several proteins only the rate constant which directly control the protein are most influential in determination of the value of maximum or minima.

**Minimum**

**Maximum**
