# Supplementary material for: Sensitivity Analysis of Intracellular Signaling Pathway Kinetics Predicts Targets for Stem Cell Fate Control
Source: PLoS Comput Biol. 2007 Jul 6;3(7):e130. doi: 10.1371/journal.pcbi.0030130 (PMC1913098; doi:10.1371/journal.pcbi.0030130)
Supplement: Supplementary file 11 [file pcbi.0030130.st001.doc]

**Supplementary Table 1**

| Rate constant symbol | Value | Description | Reference |
| --- | --- | --- | --- |
| K_1_ | 0.2 nM^-1^ Min^-1^ | LIF binding with LIF receptor | ^1-5^ |
| K_2_ | 0.0011 Min^-1^ |  |  |
| K_3_ | 6 nM^-1^ Min^-1^ | Ligand bound Receptor dimerization | ^6^ |
| K_4_ | 60 Min^-1^ |  |  |
| K_5_ | 50 Min^-1^ | JAK mediated receptor phosphorylation | ^7,8^ |
| K_6_ | 5 Min^-1^ |  |  |
| K_7_ | 3 nM^-1^ Min^-1^ | SH2 mediated Stat3 recruitment to receptor complex | ^7,9^ |
| K_8_ | 30 Min^-1^ |  |  |
| K_9_ | 30 Min^-1^ | Stat3 phosphorylation and dissociation from surface receptors | ^10^ |
| K_10_ | 0.6 nM^-1^ Min^-1^ | SOCS3 interaction with phosphorylated receptor | ^9,11^ |
| K_11_ | 30 Min^-1^ |  |  |
| K_12_ | 6 Min^-1^ | Dephosphorylation of the SOCS3-bound receptor complex | ^7,12^ |
| K_13_ | 2 Min^-1^ | Stat3 nuclear import | ^9,13-17^ |
| K_14_ | 2 Min^-1^ | Phospho-stat3 nuclear import |  |
| K_15_ | 0.04 Min^-1^ | Dephosphorylation of Stat3 in nucleus | Experimentally determined |
| K_16_ | 2 Min-1 | Stat3 nuclear export | ^11,17-19^ |
| K_17_ | 0.1 Min-1 | Phospho-Stat3 nuclear export |  |
| K_18_ | 0.3 nM^-1^ Min^-1^ | Intracellular PIAS3 interaction with cytoplasmic phosphorylated Stat3 | ^7^ |
| K_19_ | 30 Min^-1^ |  |  |
| K_20_ | 0.3 nM^-1^ Min^-1^ | Intracellular PIAS3 interaction with nuclear phosphorylated Stat3 |  |
| K_21_ | 30 Min^-1^ |  |  |
| K_22_ | 0.63 Min^-1^ | Transcription of SOCS3 mRNA | ^11,20^ |
| K_23_ | 0.28 Min^-1^ | Nuclear export of SOCS3 mRNA | ^21,22^ |
| K_24_ | 0.1 Min-1 | Degradation of SOCS3 mRNA | ^9,11,16,23,24^ |
| K_25_ | 0.01 Min-1 | SOCS3 translation |  |
| K_26_ | 0.007 Min^-1^ | SOCS3 degradation | ^25^ |
| K_27_ | 0.0263 Min^-1^ | Endogenous Stat3 phosphorylation | Experimentally determined |
| K_28_ | 0.0099 Min^-1^ | Surface receptor internalization rate constant and production rate of new receptors |  |
| K_29_/K_30_ | 0.0093 nM Min^-1^ |  |  |

**Description of rate constants**

**K_1_, K_2_:**	K_1_ and K_2_ are the forward and reverse rate constants for attachment of LIF to LIFR.Using a range of cell types, Hilton *et. al* identify two classes of LIF receptors, low- and high-affinity receptors. High affinity receptors are more prevalent and have a k_a_ of 2-8 × 10 ^8^ min^-1^M^-1^ and k_-a_ of about 0.0004 to 0.0011 min^-1^, for associate and dissociation respectively. Low affinity receptors typically have k_a_ of 3-7 × 10 ^8^ min^-1^M^-1^ and a k_-a_ of about 0.30 to 0.67 min^-1^ ^1,2^. We used a K_1_ of 0.2 min^-1^nM^-1^ and a K_2_ of 0.0011 min^-1^ corresponding to the high affinity receptor types in accordance with previous work^3-5^.

**K_3_, K_4_:**	K_3_ and K_4_ are the forward and reverse rate constants for attachment of gp130 receptor to LIFR-LIF complex. Lateral diffusion of receptors on cell surface, which influences the rate of receptor dimerization, is often a function of cell membrane interaction and influences K_3_. Asthagiri *et. al* use a typical forward binding rate constant of 6 × 10^8^ M^-1^ min ^-1^ for dimerization of ligand bound receptors^7^. In the Jak/Stat system however there is evidence of pico-molar affinity interaction between receptors of the IL-6 system, a characteristic that has been attributed to low lateral freedom on cell surface and high probability of pre-associated receptors^6^. In accordance with this observation, we used a value of 6 nM^-1^ min ^-1^ for K_3_, and a value of 60 min ^-1^ for K_4_ corresponding to similar dissociation rate constant but about 100 fold higher affinity as that reported by Asthagiri *et. al*.

**K_5_, K_6_:**	K_5_ and K_6_ are the forward and reverse rate constant of kinase phosphorylation of LIFR-GP130 complex respectively and typical values for these constants are in the range of 50 nM min ^-1^ and 5 min ^-1^ in that order^7,8^.

**K_7_, K_8_:**	K_7_ and K_8_ are based on previously published typical values for Src-Homology-2 (SH2) domain mediated intracellular protein binding to activated receptors ^7,9^.

**K_9_:**	K_9_ is the rate constant for Stat3 phosphorylation and detachment from LIFR-GP130 receptor complex. Typical range of values for this rate constant is in the order of 0.1-1 s^-1^. We used a value of 0.5 s^-1^ or 30 min^-1^ assuming that phosphorylation is the rate limiting step, and that detachment of Stat3 from the complex and JAK is subsequent to and dependant on phosphorylation^10^.

**K_10_, K_11_:**	SOCS3 phosphatase activity is captured through the rate constants K_10_ and K_11_ for SOCS3 association and dissociation from the ligand bound receptor complex of GP130 and LIFR. This is an SH2 domain mediate interaction and typical values were used for this reaction based on previous models of Jak/Stat pathway ^9,11^.

**K_12_:**	K_12_ is the rate constant for dephosphorylation of activated receptor complexes on cell surface.

A typical value of 3-6 min^-1^ has been used previously for deactivation of activate receptor complexes in the MAPK pathway^7,12^. A typical value of 6 min^-1^ was used for this rate constant.

**K_13_, K_14_:**	K_13_ and K_14_ are the rate constants for phosphorylated and unphosphorylated Stat3 nuclear import. Stat3 is actively transported into the nucleus through importins, in particular importin α3 and α5^13,14^. It is suggested that Stats mostly exist as dimers^15^. Previous differential equation models of Jak/Stat^9,16^ have used the same import rate constant which was originally use by Yamada *et. al* for Stat1^11^, however this was not experimentally determined. It is important to use experimental results for this rate constant since importin mediated nuclear import of Stat3 occurs through active transport. We use characteristic diffusion time base on observation of Watanabe *et. al* of Stat3 dynamics in living cells to determine a value of 2 min^-1^ for these rate constants^17^. It is also assumed based on the same study that nuclear retention of phosphorylated Stat3 is achieved through differential export rates from the nucleus.

**K_15_:**	Nuclear phosphatase activity which results in dephosphorylation of Stat3 is captured by rate constant K_15_.

This rate constant was determined experimentally by transient stimulation of cells with 500 pM LIF for 1 hour, followed by incubation with Jak inhibitor (as previously described) starting at time t=0. Jak inhibitor abolishes phosphorylation of Stat3 at the receptor tyrosine kinase site therefore additional phosphorylated Stat3 does not enter the nucleus. Hence only the phosphorylated Stat3 which is already present in the nucleus is detected and the rate constant for dephosphorylation can be determined by measuring the rate of decrease of nuclear phospho-Stat3. The change in concentration was determined using the trapezoidal method and the data is presented in the following plot.

Error bars represent the 10% deviation that exists due to experimental error, R^2^ is the regression value for closeness of fit and the intercept can be assumed to be zero for all practical purposes. The slope of the line of best fit, for the plot of change in concentration as a function of concentration of nuclear phospho-Stat3 represents the rate constant for dephosphorylation, and is measured to be 0.04 min^-1^. The units of concentration are arbitrary and cancel out from the calculation.

**K_16_, K_17_:**	K_16_ and K_17_ are the rate constants for nuclear export of un-phosphorylated and phosphorylated Stat3 respectively. Stat3 nuclear export is mediate through Nuclear Export Signals (NES) elements, and is dependant on CRM1, a nuclear export protein^18^. Yamada *et. al* use a value of 3 min^-1^ for nuclear export of Stat1^11^. Lam *et. al* estimate a half life of about 25 seconds for CRM1 mediated export of Parathyroid related protein (PTHrP) using Fluorescence Recovery After Photo bleaching (FRAP) of COS-1 cells^19^. This translates into a rate constant of export of about 1.7 min^-1^. In accordance with these typical values, we used a value of 2 min^-1^ for nuclear export rate constant of Stat3. Although previous differential equation models of Jak/Stat assume that only unphosphorylated Stat exits the nucleus, we included a rate constant for phosphorylated Stat3 nuclear export in order to be able to capture any interesting behaviours that may arise due to interaction with other parameters in sensitivity analysis. In accordance with previous findings which show at least an order of magnitude decrease in the rate of phosphorylated Stat3 nuclear export^17^ as compared to un-phosphorylated nuclear Stat3, we used a 20 fold lower value than that of K_16_ for this rate constant. This decrease in nuclear export of Stat3 was determined based on rise time of the activation profile of Stat3.

**K_18_, K_19_, K_20_, K_21_:**	K_18_ and K_19_ are rate constants for cytoplasmic PIAS3 interaction with Stat3. K_20_ and K_21_ are rate constants for nuclear PIAS3 interaction with Stat3.Typical values for intracellular adapter protein interactions were used for these rate constants based on work of Asthagiri *et. al*^7^. A value of 0.3 nM^-1^min^-1^ was used for K_18_ and 30 min^-1^ was used for K_19_, the same values were used for K_20_ and K_21_ respectively for nuclear PIAS3 interaction with nuclear Stat3.

**K_22_:**	K_22_ is the rate constant for SOCS3-mRNA production.

Rate of mRNA production in the nucleus is dependant on at least three events, attachment of RNA polymerase to DNA, opening of DNA and transcription, and finally detachment of mRNA from the complex. Previous studies show that the rate limiting step (the slowest step) has a value of about 0.0105s^-1^ for mRNA production^20^. By using this general value as a maximal production rate constant, and assuming the active DNA segment to be bound to nuclear Stat3, we obtain a value of 0.63 min^-1^ for this rate constant. This value is about one quarter of the maximal value of Stat1 mediated Socs1 mRNA production based on the development of Yamada *et. al*^11^ for the Stat1 system. For simplicity a first order kinetics were assumed.

**K_23_:**	K_23_ is the rate constant for export of SOCS3 mRNA from the nucleus. Audibert *et. al*^21^ indicate a mRNA nuclear half life of about 2.5-4.5 minutes in HeLa cells for average size mRNAs and nuclear dwelling time of about 4 minutes, which can be appropriately captured using first order kinetics^21^. In accordance with these typical values we use a value of 0.28 min^-1^ for SOCS3 nuclear export rate constant. This value is by no means exact; however it is in the correct order of magnitude. Furthermore nuclear export rate is of little significance to the overall localization of the mRNA since the rate of cytoplasmic degradation is usually smaller^22^, therefore it is assumed that this typical value will be sufficient for our analysis. This assumption was later verified by experimental determination of SOCS3 induction kinetics by LIF stimulated Stat3 activation in this study.

**K_24_, K_25_:**	SOCS3 mRNA is a relatively unstable mRNA with a half-life much shorter than 30 minutes^23,24^. In accordance with this we assumed a typical value of 0.1 min^-1^ for K_24_; this corresponds to an mRNA half life of about 7 minutes. For the rate constant K_25_ corresponding to translation of SOCS3, a typical value of 0.01min^-1^ was used in accordance with previous models^9,11,16^.

**K_26_:**	SOCS3 degradation is assumed to be first order with the rate constant K_26_. Half life of SOCS3 protein is about 1.6 hours^25^. Therefore a good estimate of K_26_ is about 0.0072 min^-1^.

**K_27_:**	In order to account for endogenous phosphorylation of Stat3 a first order reaction which converts Stat3 to phosphorylated Stat3 was used with a rate constant K_27_. K_27_ was adjusted to match the endogenous level of phosphorylated Stat3. This level of endogenously phosphorylated Stat3 is not attributable to exogenous LIF because it exists in the absence of LIF in the media. K_27_ is the only rate constant which was adjusted in this model so that the initial level of normalized phosphorylated Stat3 (before LIF stimulation) in the model matches experimental results.

**K_28_:**	K_28_ is the rate constant for internalization of surface receptors. Internalization of all surface receptors is modeled using first order kinetics. This rate constant was determined experimentally by flow cytometry analysis of surface receptor expression. The half life of surface receptors was found to be about 70 minutes.

**K_29_:**	K29 is the rate of receptor production for both LIFR and GP130. This rate was determined based on K28 and the number of receptors on cell surface, such that steady state level of receptors remains constant. In the special case of the GP130 over-expression studies we designated GP130 receptor production with the rate constant K30 in order to show that this rate constant was changed independently of LIFR production rate.

**References:**

1.	Hilton DJ, Nicola NA. Kinetic analyses of the binding of leukemia inhibitory factor to receptor on cells and membranes and in detergent solution. J Biol Chem 1992;267(15):10238-47.

2.	Hilton DJ, Nicola NA, Metcalf D. Distribution and comparison of receptors for leukemia inhibitory factor on murine hemopoietic and hepatic cells. J Cell Physiol 1991;146(2):207-15.

3.	Godard A, Heymann D, Raher S, Anegon I, Peyrat MA, Le Mauff B, Mouray E, Gregoire M, Virdee K, Soulillou JP and others. High and low affinity receptors for human interleukin for DA cells/leukemia inhibitory factor on human cells. Molecular characterization and cellular distribution. J Biol Chem 1992;267(5):3214-22.

4.	Bitard J, Daburon S, Duplomb L, Blanchard F, Vuisio P, Jacques Y, Godard A, Heath JK, Moreau JF, Taupin JL. Mutations in the immunoglobulin-like domain of gp190, the leukemia inhibitory factor (LIF) receptor, increase or decrease its affinity for LIF. J Biol Chem 2003;278(18):16253-61.

5.	Viswanathan S, Benatar T, Rose-John S, Lauffenburger DA, Zandstra PW. Ligand/receptor signaling threshold (LIST) model accounts for gp130-mediated embryonic stem cell self-renewal responses to LIF and HIL-6. Stem Cells 2002;20(2):119-38.

6.	Schroers A, Hecht O, Kallen KJ, Pachta M, Rose-John S, Grotzinger J. Dynamics of the gp130 cytokine complex: a model for assembly on the cellular membrane. Protein Sci 2005;14(3):783-90.

7.	Asthagiri AR, Lauffenburger DA. A computational study of feedback effects on signal dynamics in a mitogen-activated protein kinase (MAPK) pathway model. Biotechnol Prog 2001;17(2):227-39.

8.	Linderman JJ LDA. Receptors: models for binding, traficking and signaling; 1993. 361 p.

9.	Singh A, Jayaraman A, Hahn J. Modeling regulatory mechanisms in IL-6 signal transduction in hepatocytes. Biotechnol Bioeng 2006.

10.	Haugh JM, Lauffenburger DA. Analysis of receptor internalization as a mechanism for modulating signal transduction. J Theor Biol 1998;195(2):187-218.

11.	Yamada S, Shiono S, Joo A, Yoshimura A. Control mechanism of JAK/STAT signal transduction pathway. FEBS Lett 2003;534(1-3):190-6.

12.	Schoeberl B, Eichler-Jonsson C, Gilles ED, Muller G. Computational modeling of the dynamics of the MAP kinase cascade activated by surface and internalized EGF receptors. Nat Biotechnol 2002;20(4):370-5.

13.	Liu L, McBride KM, Reich NC. STAT3 nuclear import is independent of tyrosine phosphorylation and mediated by importin-alpha3. Proc Natl Acad Sci U S A 2005;102(23):8150-5.

14.	Ushijima R, Sakaguchi N, Kano A, Maruyama A, Miyamoto Y, Sekimoto T, Yoneda Y, Ogino K, Tachibana T. Extracellular signal-dependent nuclear import of STAT3 is mediated by various importin alphas. Biochem Biophys Res Commun 2005;330(3):880-6.

15.	Braunstein J, Brutsaert S, Olson R, Schindler C. STATs dimerize in the absence of phosphorylation. J Biol Chem 2003;278(36):34133-40.

16.	Zi Z, Cho KH, Sung MH, Xia X, Zheng J, Sun Z. In silico identification of the key components and steps in IFN-gamma induced JAK-STAT signaling pathway. FEBS Lett 2005;579(5):1101-8.

17.	Watanabe K, Saito K, Kinjo M, Matsuda T, Tamura M, Kon S, Miyazaki T, Uede T. Molecular dynamics of STAT3 on IL-6 signaling pathway in living cells. Biochem Biophys Res Commun 2004;324(4):1264-73.

18.	Bhattacharya S, Schindler C. Regulation of Stat3 nuclear export. J Clin Invest 2003;111(4):553-9.

19.	Lam MH, Henderson B, Gillespie MT, Jans DA. Dynamics of leptomycin B-sensitive nucleocytoplasmic flux of parathyroid hormone-related protein. Traffic 2001;2(11):812-9.

20.	Arkin A, Ross J, McAdams HH. Stochastic kinetic analysis of developmental pathway bifurcation in phage lambda-infected Escherichia coli cells. Genetics 1998;149(4):1633-48.

21.	Audibert A, Weil D, Dautry F. In vivo kinetics of mRNA splicing and transport in mammalian cells. Mol Cell Biol 2002;22(19):6706-18.

22.	Cao D, Parker R. Computational modeling of eukaryotic mRNA turnover. Rna 2001;7(9):1192-212.

23.	Sasaki A, Inagaki-Ohara K, Yoshida T, Yamanaka A, Sasaki M, Yasukawa H, Koromilas AE, Yoshimura A. The N-terminal truncated isoform of SOCS3 translated from an alternative initiation AUG codon under stress conditions is stable due to the lack of a major ubiquitination site, Lys-6. J Biol Chem 2003;278(4):2432-6.

24.	Wormald S, Zhang JG, Krebs DL, Mielke LA, Silver J, Alexander WS, Speed TP, Nicola NA, Hilton DJ. The comparative roles of suppressor of cytokine signaling-1 and -3 in the inhibition and desensitization of cytokine signaling. J Biol Chem 2006;281(16):11135-43.

25.	Heinrich PC, Behrmann I, Haan S, Hermanns HM, Muller-Newen G, Schaper F. Principles of interleukin (IL)-6-type cytokine signalling and its regulation. Biochem J 2003;374(Pt 1):1-20.
